# Social robots counselling in community pharmacies – Helping or harming? A qualitative study of pharmacists' views

**DOI:** 10.1016/j.rcsop.2024.100425

**Published:** 2024-02-24

**Authors:** Sara Rosenberg, Malin Andtfolk, Susanne Hägglund, Mattias Wingren, Linda Nyholm

**Affiliations:** a Department of Caring Science, Faculty of Education and Welfare studies, Åbo Akademi University, Vaasa, Finland; b Pharmaceutical Sciences Laboratory, Faculty of Science and Engineering, Åbo Akademi University, Turku, Finland; c Experience Lab, Faculty of Education and Welfare studies, Åbo Akademi University, Vaasa, Finland; d Department of Caring and Ethics, Faculty of Health Sciences, University of Stavanger, Stavanger, Norway

**Keywords:** Social robot, Medication safety, Medication counselling, Community pharmacy, Pharmacist

## Abstract

**Background:**

Welfare technological solutions such as social robots attempt to meet the growing needs of the healthcare sector. Social robots may be able to respond to the shortage of pharmaceutical personnel at community pharmacies. However, there is a lack of previous studies regarding the use of social robots for medication counselling purposes in a pharmacy setting.

**Objectives:**

The objective of this qualitative study was to explore pharmacists' views on the potential role of social robots in medication counselling.

**Methods:**

Pharmacists, purposively sampled based on having recent experience of counselling customers in community pharmacies in Finland, first acted as customers interacting with the social robot in a simulated setting, before taking part in a focus group where their perspectives were explored. The focus group discussions were conducted in October and November 2022. The qualitative data was transcribed and analysed using reflexive thematic analysis.

**Results:**

The number of participants was eight in total. A main theme of how the robot may either help or harm concerning medication safety within a pharmacy setting was identified. The six sub-themes found, according to pharmacists' views on robot counselling in community pharmacies, are context, digital competence, customer integrity, interaction, pharmacists' professional role and human skills.

**Conclusions:**

According to the study findings, pharmacists experience that the social robot can offer a potential complement to a human pharmacist. The robot is seen as beneficial with respect to certain customer groups and in the light of personnel shortages, and may in the future add to trust, equality, freedom of choice and multilingualism, among other things, in the customer service situation at community pharmacies, thus improving medication safety.

## 1 Introduction

According to Finnish legislation,^1^ a pharmacist is obliged to give medication counselling to pharmacy customers by informing and guiding them on the appropriate use of relevant medication, with the aim of confirming successful pharmacotherapy and supporting medication and patient safety. Furthermore, customers are to be informed of the price of pharmaceuticals including information on the cheapest alternative available at the time of dispensing. As shortcomings in pharmacotherapy implementation may jeopardise medication and patient safety, successful high-quality medication counselling is essential since it can prevent medication errors and add to a more efficient use of resources overall.^2^

Safe and successful pharmacotherapy can be said to consist of two sub-areas, namely pharmaceutical and medication safety.^3^ In short, pharmaceutical safety ascribes to the safety of a medicinal product, whereas medication safety ascribes to the safety of the drug treatment itself. Medication errors are one of the biggest yet avoidable challenges in healthcare.^4^ According to the World Health Organization (WHO), the cost of medication errors at a global level has been estimated at USD 42 billion annually. Medication errors can occur because of human factors such as personnel shortages, fatigue, and/or poor environmental conditions which in turn affect the prescription, dispensing and administration of medicines.

The factors potentially leading to medication errors in community pharmacies are affected by compromised workforce resilience and job dissatisfaction among pharmacy professionals. Job satisfaction among pharmacists has been shown to increase with their opportunity to continue professional development.^5^ On the contrary, job satisfaction may be compromised by a sense of dissatisfaction with the professional environment^6^ and with management.^7^ During the recent pandemic, pharmacy professionals experienced increased levels of workload and increased psychosocial burden.^8^, ^9^, ^10^, ^11^, ^12^ As a part of the global shortage of healthcare workers,^13^ the acknowledgement of these issues is relevant in the field of community pharmacy and pharmacy education.^14^

Currently, there is also a vast shortage of personnel within the social and healthcare services in Finland, including pharmacists in community pharmacies.15., 16., 17. This shortage is due to various reasons such as a growing number of pharmacies, an increased number of pharmacists working clinically in hospitals and the content of the work in community pharmacies not meeting the expectations of pharmacists.^16^ Alongside this, the universities educating pharmacists do not have the opportunity to quickly and independently increase the amount of pharmacy students due to strained budget proposals and a shortage of teaching resources.^16^

As an attempt to meet the growing needs of healthcare, welfare technological solutions in the form of social robots, for example, have been proposed.^18^ However, the utilisation of social robotics is still modest within the welfare sector.^19^ According to The Handbook of Robotics,^20^ the definition of a social robot reads ‘Social robots are designed to interact with people in human-centric terms and to operate in human environments alongside people. Many social robots are humanoid or animal-like in form, although this does not have to be the case. A unifying characteristic is that social robots engage people in an interpersonal manner, communicating and coordinating their behaviour with humans through verbal, nonverbal or affective modalities’. Furthermore, social robots are often regarded as so-called technological fixes developed to solve pressing social problems of a non-technical nature.^21^

In the context of human-robot interaction (HRI), social robots assume a distinctive position and are categorised as engaging in ‘proximate interaction’, where humans and robots interact on a peer-to-peer or companionable basis.^22^ Numerous developers of social robots have strived to imbue their creations with human-like qualities. However, they have been cautious not to make them mimic human appearance or movements too closely. This strategic balance is aimed at avoiding the unsettling feeling associated with robots that look almost human i.e., the ‘uncanny valley’.^23^^,^^24^ Although a human-like appearance is a strong indicator that a social robot can engage in social interactions, it also increases the risk of not meeting the high expectations regarding the quality of these interactions.^25^^,^^26^

A mapping of previous studies regarding the use of social robots in medication processes has been conducted recently.^27^ Within this mapping, studies were found on the attitudes and experiences of users concerning the utilisation of such robots for monitoring and assessing their medication adherence in real home settings, for example.^28^^,^^29^ One study found that the medication adherence of patients suffering from chronic obstructive pulmonary disease (COPD) may be improved by a robot reminding them to take their medication and recording adherence frequently – many times a day.^28^ For elderly patients with mild cognitive impairments (MCI), a pipeline for robot assistance concerning medication adherence, including action, perception and cognition skills, has shown promising results.^29^ A recent review of artificial intelligence in pharmacy practice focuses on dispensing robots and robotic technology for preparation and tracking of medications.^30^ The use of social robots for medication counselling purposes in a pharmacy setting has, however, not been studied previously.

### 1.1 Aim of the study

The aim of this study was to explore the views of pharmacists regarding a social robot giving medication counselling in community pharmacies in relation to medication safety.

## 2 Methods

### 2.1 Intervention description

**Fig. 1:**
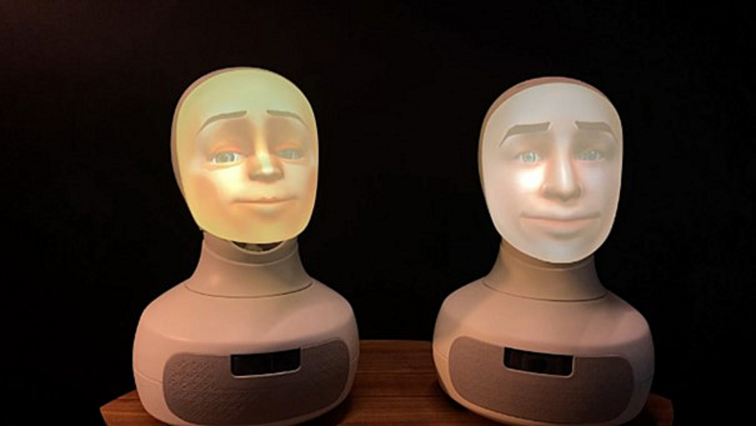
The social robot Furhat (re-printed with permission).^32^

**Fig. 2:**
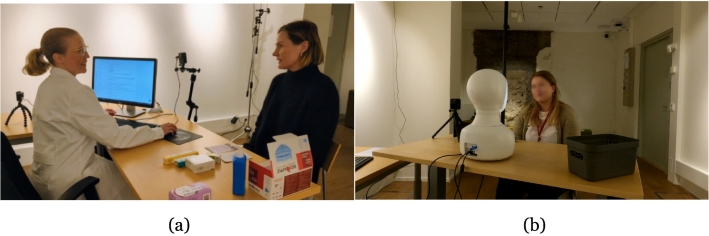
Pharmacist (a) and social robot (b) interacting with potential customers in a simulated pharmacy setting (re-printed with permission).

As part of the PharmAInteraction project in Ostrobothnia, Finland,^31^ aiming to co-design and iteratively test a robot application to be used for medication counselling purposes in community pharmacies, the study utilised a social robot called Furhat (Fig. 1). This robot is developed by Furhat Robotics and has been called *the world's most advanced social robot*.^32^ It was selected as its appearance, size, and capacity to make conversation in several different languages were seen to be in alignment with the research aim. The application used was designed to enable the robot to act as a Swedish-speaking pharmacist, providing medication counselling related to emergency contraceptive pills according to country-specific legislative requirements,^33^ in order to increase medication safety. As a first step in the application design process, the participating pharmacists met potential customers in a simulated pharmacy setting on a university campus human-technology interaction laboratory, in order for the researchers to deepen their contextual understanding of the specific customer service situation (Fig. 2a). Hence, the robot application was developed based on the legislative requirements and customers' needs. The detailed application design process is described elsewhere.^34^

### 2.2 Study design

The qualitative methodology selected for the study was focus group discussions. Qualitative discussions of this kind were considered to be a suitable method for data collection when attempting to elucidate the views and attitudes of participants on a novel subject area and identifying themes related to it.^35^, ^36^, ^37^ The strength of the focus group discussion lies within the interaction between the participants sharing their knowledge and experiences, potentially bringing about views that would be left out in individual interviews. Also, participants may feel more comfortable giving critical feedback as part of a group. The ideal focus group size was three participants per group, i.e. small focus groups with participants possessing specialised knowledge.^38^

### 2.3 Participant selection and recruitment

Participants were recruited through purposive sampling by contacting pharmacists who work (or have recently worked) at community pharmacies in Finland. The first author (SR) contacted participants by phone/email to invite them to participate in the study. The eligibility criteria were that the pharmacists should currently be, or have in the past year been, engaged in medication counselling tasks at a community pharmacy. The pharmacy education in Finland includes a three-year bachelor's degree and a five-year master's degree.^39^ Both have medication dispensing certifications and hence the term pharmacist will be used here including both degrees unless otherwise specified.

### 2.4 Data collection

In the second step of the project, following the application design process, the application prototype was tested by potential pharmacy customers in the simulated pharmacy setting for the evaluation of user experiences concerning the HRI.^40^ As a part of step two, the current study commenced with the participating pharmacists acting as pharmacy customers, individually interacting with the Furhat robot in the simulated pharmacy setting (Fig. 2b). The participants were instructed to present a request to buy an emergency contraceptive pill with somewhat varying scenarios (Table 1). The individual interactions were followed by the focus group discussions.

**Table 1:** The two scenarios used in the participant-robot interaction. ⁎

Scenario 1 (two days)	Scenario 2 (four days)
Imagine that you are visiting the pharmacy to buy the emergency contraceptive pill. You tell the pharmacist that you had unprotected sex two days ago. As a customer, you may want to find out information about the medicine and you may ask the pharmacist questions as well. When you have received the medicine, you can leave the pharmacy.	Imagine that you are visiting the pharmacy to buy the emergency contraceptive pill. You tell the pharmacist that you had unprotected sex four days ago. As a customer, you may want to find out information about the medicine and you may ask the pharmacist questions. When you have received the medicine, you can leave the pharmacy.

⁎ The reason for using two distinct scenarios was to test the robot application with respect to counselling of the two different emergency contraceptive pill APIs (levonorgestrel and ulipristal acetate) available on the market in Finland having different time windows of effect.

Construction of the interview guide for the focus group discussions was executed by four of the researchers (SR,MA,MW,LN). The first draft was put together by one of the researchers (MA), subsequently discussed and further polished by three (SR,MA,MW), and finally tested and approved for use in conjunction with the first focus group discussion by a fourth (LN). This testing did not result in revisions to the guide, and thus findings from the discussion in question were included in the study results. The interview guide was based on the aim of the study together with the mapping of previous research regarding the use of social robots in medication processes,^27^ and the simulations between pharmacists and potential customers in real life in the simulated pharmacy setting explained above.^34^ The questions in the interview guide were the same for all focus groups and dealt with the following subject areas: participant background (work and technology experience including previous experience of social robots), HRI in general, HRI with respect to emergency contraception, HRI and safety, and future prospects (see Appendix A).

The three focus group discussions were conducted during October and November 2022. The discussions were conducted face-to-face by one of the authors (LN) and lasted from approximately 55 to 69 min. The interviewer has extensive experience in interviewing and had no established relationship with any participant. The post-test discussions were conducted in connection with the simulated pharmacy setting where the interaction with the robot had taken place. This was a neutral and undisturbed location to make the participants feel comfortable and relaxed. The discussions were conducted in Swedish, as this is the native language of the interviewer and all but one of the participants. This non-native Swedish-speaking participant was also fluent in Swedish and had the option of using their own native language which was Finnish. The interviewer offered the participants of each group the opportunity to speak at length and discuss their views on the subject matter. The discussions were audio-recorded, and verbatim transcription of the recorded material was conducted afterwards by three of the authors (SR, MA, LN). All collected data was pseudonymised.

### 2.5 Data analysis

**Fig. 3:**
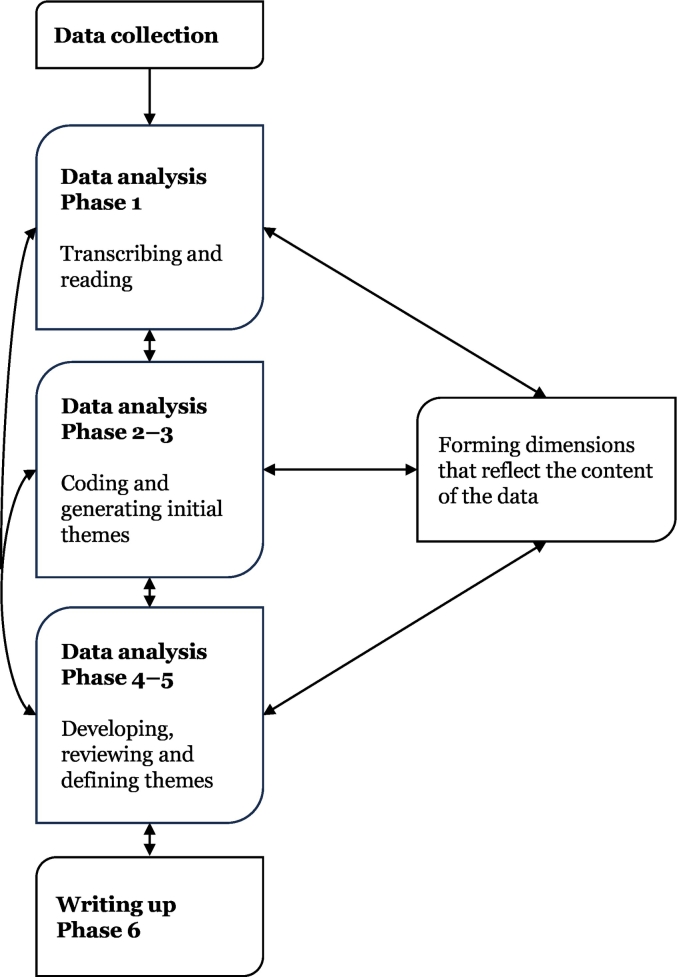
Steps of the reflexive TA scheme according to Braun and Clarke.^41^ Reworked figure based on model by Lantz.^42^

The data was analysed manually and in an inductive manner, with theme development originating from within and motivated by the data content, using the flexible reflexive thematic analysis (TA) scheme by Braun and Clarke (Fig. 3).^41^ After finalisation of the focus group discussions, these were manually transcribed by three of the authors (SR, MA, LN). Following transcription, the analysis process was continued by the transcripts being read and re-read multiple times according to phase 1 in Fig. 3 (SR, MA, LN). The same three authors then analysed the transcribed data together, leading to categorisation of codes, through initial themes to themes according to phases 2–3 of the analysis scheme (Fig. 3). Finally, themes were refined and confirmed as being reflective of the data content through discussions including the whole research team in accordance with phases 4–5 (Fig. 3). In the iterative analysis process utilised here, moving back and forth between the steps of analysis as described in Fig. 3, the research team worked with the data until satisfactory dimensions that were reflective of the same had been achieved. The writing process (phase 6, Fig. 3) was ongoing throughout the analysis.

### 2.6 Ethical considerations

The guidelines for good scientific praxis as described by TENK^43^ and the World Medical Association's Declaration of Helsinki^44^ have been followed during this research. The participants were given information about the study, and the purpose of the study was explained. Participants were guaranteed confidentiality, informed that participation was voluntary, and that they could withdraw from the study at any time. Prior to the focus group discussions, all the participants had provided their written informed consent. With reference to the Finnish National Board on Research Integrity TENK,^45^ an ethical review statement from a human sciences ethics committee is not warranted for this study design.

### 2.7 Findings

The number of participants in the three focus group discussions conducted was eight in total (*n*_*total*_ = 3 + 3 + 2 = 8). Due to illness, one group fell short of a participant on the day of the discussion and therefore consisted of only two participants instead of the desired three. Participant characteristics and detailed discussion durations are presented in Table 2.

**Table 2:** The number of participants per focus group (*n*), participant characteristics, scenario, and detailed discussion durations.

	Focus group 1 (*n* = 3)	Focus group 2 (*n* = 3)	Focus group 3 (*n* = 2)
Gender			
Female *n* (%)	3 (100)	3 (100)	2 (100)
Male *n* (%)	0 (0)	0 (0)	0 (0)
Median age (y)	46	50	35
Median counselling experience (y)⁎	19	24	12
Current place of work (*n*)	Commun. pharm. (2)	Commun. pharm. (2)	Commun. pharm. (2)
	Industry (1)^⁎⁎^	Hospital pharm. (1)^⁎⁎^	
Experience of social robots	None	None	None
Scenario	1	2	1
Interview duration	55 min 50 s	67 min 26 s	68 min 39 s

⁎ y_max_ = 31, y_min_ = 3. Pharmacists have worked within a community pharmacy in the past year before the focus group discussions and have several years of experience of working with medication counselling tasks at community pharmacies.

The sum result of the analysis is given in Table 3, followed by a more detailed description including significant quotes extracted from the data. The six sub-themes: context, digital competence, customer integrity, interaction, pharmacists' professional role and human skills, are all somehow connected to the main theme of how the robot may either help or harm concerning medication safety. Together they offer an overall insight into pharmacists' views on using a social robot for medication counselling purposes in community pharmacies.

**Table 3:** A sum result of the analysis describing the main theme and the six sub themes found.

	Helping	Harming
Context	The pharmacy context adds to a sense of trust and quality with respect to the information provided	An alternative context such as a grocery store without pharmaceutical personnel constitutes a risk
Digital competence	The choice of the emergency contraceptive pill is based on a presumed higher digital competence of the potential customers	Customer qualities such as age and disabilities may affect digital competence and add to vulnerability
Customer integrity	An advantageous choice if the customer wants to stay anonymous due to small community and/or fear of being judged	The loudness of the robot and the delicacy of the information calls for boundaries, but which are not too obvious in nature
Interaction	The robot's capacity to speak several different languages and its human-like appearance provides value	A sense of inability to interrupt the robot to ask additional questions and the high speed of information given
Pharmacists' professional role	An aid for pharmacists with respect to personnel shortages and potentially aggressive customers, among other things	A risk of pharmacists being replaced by robots. The information quality could be improved by connecting the robot to relevant databases
Human skills	Offers a potential complement to the human pharmacist and may be used to improve the service level by offering the customer a choice in service provider	Human pharmacists can identify subtle signs relevant to communication, such as body language and untrustworthiness

1. CONTEXT: The pharmacy context represents professionality and adds to trust in the information provided, and thereby to medication safety, when using a robot.

Early on in the focus group discussions, pharmacists brought up the relevance of the pharmacy context with respect to medication safety when using the robot for medication counselling purposes, ‛… *people trust pharmacies, so I would say that I trust the robot that is in the pharmacy more than the robot that is in Citymarket* [grocery store]’ (Pharmacist 8). The scenario of medicines potentially being sold in grocery stores in Finland in the future was generally criticised as it is feared this will become a medication safety issue due to the lack counselling available in that alternative environment. Also, the risk of social robots becoming the sole providers of medication counselling in grocery stores was raised, ‛… *so do grocery stores exploit this then?*’ (Pharmacist 2). Pharmacists felt that customers can trust the information given by the robot, specifically when it is provided in a pharmacy. However, they questioned the completeness of the information content given to them by the robot during the current interaction, ‛… *it doesn't say anything inaccurate either, but it could be that it doesn't give all the information but the correct information*’ (Pharmacist 7).

2. DIGITAL COMPETENCE: The digital competence of the customer affects medication safety when using a robot.

With regard to the novelty of social robots in the pharmacy context, pharmacists raised their concerns about the digital competence of customers with respect to medication safety. The choice of the emergency contraceptive pill for the current study is seen as relevant with respect to the target group being young women, a group of customers considered to be more digitally competent than the average customer as a result of their age; thus being more competent to use the robot, ‛*I think the idea was good because it is a drug that needs that extra information and a drug that is aimed at younger women*’ (Pharmacist 3). On the other hand, young people not being critical enough of the quality of the information handed to them via different technological solutions such as a robot is seen as a matter of concern.

Pharmacists also raised the relevance of the robot being easy enough to use with respect to potential disabilities among the customer group, and an exit button on the robot, which would call for a human pharmacist, was requested, ‛*Poor hearing, poor eyesight … the simplest would be that there was only one red button in the middle*’ (Pharmacist 7). Overall, it is acknowledged that digital competence is relevant in securing medication safety since health technological solutions are becoming more common. Digital competence was also thought to make such solutions more attractive to some occupational groups, ‛… *an engineer who is really like: Oh! A robot. I get to talk to it*’ (Pharmacist 6).

3. CUSTOMER INTEGRITY: Ensuring customer integrity/anonymity adds to a sense of security/accessibility, and thereby to medication safety, when using a robot.

Arising from the discussion on context, another issue highlighted by the pharmacists was one of the importance of securing customer integrity. The robot is experienced as being quite loud and with a mechanical voice, which is seen as problematic, ‛*You can't tell the robot to whisper if aunty were to come up behind you*’ (Pharmacist 3). The delicate nature of buying the emergency contraceptive pill is also mentioned in terms of this issue, adding to its importance. Hence, the robot being placed in a separate room or at least having walls around it was argued to add to a sense of security for the customer and the receptiveness of the information given, ‛… *some kind of booth or against the wall, that you are alone there with it and not out there in the middle of the room*’ (Pharmacist 7). Consequently, however, pharmacists pointed out that it should not be obvious to others which medicine the customer is buying, i.e. the robot should not give information on only one kind of medicine. Also, shortcomings in data protection when using AI-based solutions like social robots was raised as a matter of concern.

Especially in small towns and communities, the use of a robot providing medication counselling concerning medicines of a potentially delicate nature was seen as a great opportunity among the pharmacists, ‛*If it is the case that there are very few people at the pharmacy and there is nothing else … in small towns*' (Pharmacist 1). This is also seen as beneficial for shy and/or embarrassed customers and for those afraid of being judged by another human being, ‛*It's an advantage with a robot, because it doesn't matter what you're wearing*’ (Pharmacist 1). In such cases it was speculated whether the information is more likely to reach the customer when given by a robot instead of a human.

4. INTERACTION: Various issues regarding the interaction between customer and robot affect medication safety.

From a medication safety point of view, pharmacists had many concerns regarding the interaction between customers and robots. When meeting the robot for the first time, some experienced it as something that made them nervous and they had a difficult time concentrating on anything but the robot's appearance, ‛*It is also an unusual situation. Even though I'm a pharmacist, I can feel some kind of nervousness when I'm going to meet a robot*’ (Pharmacist 3). Others were surprised that the robot was much more human-like and trustworthy than expected, *‛I thought it would be some kind of toy, so it was better than I thought*’ (Pharmacist 8). The speed at which the robot spoke was considered too fast for the customer to be able to remember everything, and several questioned the impossibility to interrupt the robot whenever they wanted to ask a question. Many concerns were also expressed regarding the robot's ability to understand the customer correctly, ‛*I don't know how the robot took my answer*’ (Pharmacist 7).

The robot's ability to speak many different languages was considered a major advantage in comparison to a human pharmacist in ensuring medication safety in situations where the customer and the pharmacist have different native languages, ‛*They may not speak the same language, they speak* via *Google Translate or something*’ (Pharmacist 4). However, some concerns were raised regarding the robot's inability at this point to change language in the middle of the counselling situation.

5. PHARMACISTS' PROFESSIONAL ROLE: Impact of the robot on the professional role of pharmacists in light of medication safety issues.

Pharmacists expressed concerns about the risk of them being replaced by the robot, even though they acknowledged the potential of such novel solutions in terms of the shortage of personnel in community pharmacies, ‛*What about the customers who are already saying that soon only the robot will be left here?*’ (Pharmacist 8). In questioning shortcomings in the interaction between the customer and the robot, the pharmacists speculated that the robot would become more reliable and comparable to a real human pharmacist if connected to relevant databases, ‛*Can you modify it later, so that you have to show your medical insurance card and it can somehow read it?*’ (Pharmacist 6). They believe that most customers would trust the robot, but they were also worried about the risk of technical problems.

The great potential of the information given in the counselling situation reaching the customer and thereby leading to medication safety was found with respect to customers who have the habit of rejecting medication counselling due to being in a hurry and/or ignorance, ‛… *they come to the shelf very quickly and go to the cash register, so you don't have time to say anything … You don't want to be rude even though they are rude … maybe it wouldn't be that dangerous, and they would be curious as to what the robot wants to say*’ (Pharmacist 2). In this regard, pharmacists experienced that the robot may also offer an aid for them concerning hostile and aggressive customers, diminishing their psychosocial burden at the workplace, ‛… *if the customer has started screaming … psychologically bullying the moment they walk in the door. I would gladly send such a customer to the robot … the robot is neutral, and it can be used by an angry customer*’ (Pharmacist 3). On the other hand, comments about humans being more emphatic in such situations were likewise brought forward.

6. HUMAN SKILLS: A human pharmacist possesses skills that the robot lacks for ensuring medication safety.

In comparing the human-human vs human-robot interactions, pharmacists stated that the robots lack of body language is a relevant shortcoming in the medication counselling situation, ‛*I sat there and waited to get to that a-ha, now it's done, and at one point I was going to start talking but it continued. And as it didn't have body language* …’ (Pharmacist 7). They also found that it was easier to withhold information from or lie to the robot than a human, and that a human pharmacist can read between the lines and ask the right questions, ‛… *medicines with an age limit. I can go there and say I'm twenty-five even though I'm fifteen … you learn to ask certain questions*' (Pharmacist 6).

Pharmacists also feel that it is important that the customer gets to choose who will give them the medication counselling, that the robot is not a suitable option to all, ‛*I think of my grandmother. If someone had told her that they didn't have time now, take this robot, then I'd probably see her walking out of the pharmacy*’ (Pharmacist 6). Furthermore, in case of the robot being used for medication counselling purposes in community pharmacies in the future, they argued that a human pharmacist always needs to be available to take over if the robot fails or if the customer asks for it. All in all, pharmacists found humans to be superior in ensuring medication safety compared to a robot. They found that the robot may offer a complement but that it cannot replace a human pharmacist, ‛*If you maybe developed it a little more. But it is probably difficult. A human being cannot be fully replaced*’ (Pharmacist 4).

## 3 Discussion

The present study examined the views of pharmacists regarding a social robot providing medication counselling in community pharmacies in relation to medication safety. A main theme of how the robot may either help or harm concerning medication safety within a pharmacy setting was identified. The six sub-themes found together with this main theme depict some prerequisites for robot counselling as identified by pharmacists and fulfil the aim of the study. These sub-themes are context, digital competence, customer integrity, interaction, pharmacists' professional role and human skills. The study findings and how they may be used to improve medication safety when using a social robot for medication counselling are discussed below.

### 3.1 Pharmacy context and trust

The pharmacy context is seen by participating pharmacists as a relevant factor in ensuring medication safety if a robot were to be used for medication counselling, as this specific context is believed to lead to more trust in the information provided. Within this discussion, being able to trust the information provided by a robot is likely to be bound to the social category, the pharmacy, within which the robot functions and the conferred trust given to the robot acting as a pharmacist. As described by Gregory and co-workers in their study on how patients develop trust in community pharmacists,^46^ conferred trust follows from external social cues such as status or positional authority. A poll performed on assignment by the Association of Finnish Pharmacies,^47^ shows that no less than 95% of the Finnish population is content with the services provided by pharmacies, which may be argued to support the perceived high degree of trust in the pharmacy context. Consequently, this adds to the requirement level of the robot and its ability to offer medication counselling without jeopardising medication safety.

On the contrary, the robot alone giving medication counselling within an alternative context such as a grocery store is seen by pharmacists to diminish trustworthiness and thereby negatively affect medication safety. They express a concern about this scenario potentially happening in the future in Finland because of the current political climate in the country and discussions on deregulation of the pharmacy field, potentially enabling sales of some over-the-counter (OTC) medicines outside pharmacies.^48^ In terms of medication safety, this concern about medicines being sold without pharmacy professionals being present as expressed by the pharmacists in the current study can be seen as called for in the light of discussions in the Nordic countries on paracetamol poisonings and the elevated use of nicotine supplements, for example.^49^^,^^50^

### 3.2 Digital competence and equality

The digital competence of pharmacy customers is another issue of concern when using a social robot for medication counselling. Pharmacists identify the type of medicine that the robot offers counselling on together with the occupation of the customer to be of relevance with respect to the customers digital competence. In this study, the emergency contraceptive pill was chosen by the research team, as its customer group is largely expected to consist of younger women. However, as stated by Yates,^51^ it should not be forgotten that inequality also limits the experiences of technology among young people. For example, regarding data literacy, their research findings show that it is not uniformly high among the young in contrast to what is easily assumed, including by the pharmacists in this study. In addition to the question of customer age, the participating pharmacists raise that occupational groups such as engineers may be more eager to be counselled by a robot due to their presumed higher digital competence. Overall, within this discussion it can be stated that the digital competence of the Finnish population is relatively high, as 79% have at least basic skills.^52^

According to one definition of digital competence,^53^ it *involves the confident and critical use of information and communications technology (ICT) for employment, learning, self-development and participation in society*. In line with this definition, while trying not to generalise too much, it can be speculated that some customer groups may well possess an advantage over others in using social robots for medication counselling purposes due to their higher digital competence. Shortcomings regarding digital competence not only due to age but also because of different kinds of disabilities is likewise found as relevant by the pharmacists with respect to the social robot in a pharmacy setting. They emphasise the importance of the robot being designed so that it is easy to use for all that want to use it. In the light of the United Nations (UN) social development goals,^54^ no. 3 *Good Health and Well-being* and no. 10 *Reduced Inequality*, it can be argued that the development of health technological devices such as the social robot in medication counselling should consider the safe and equal serving of all customer groups in order for medication safety to be ensured.

### 3.3 Customer integrity and delicate situations

In pharmacy practice, ensuring integrity for the customer is of utmost importance as the pharmacists' code of ethics state that *‛A pharmacist promotes the good of every patient in a caring, compassionate and confidential manner’*.^55^ It is therefore not surprising that the pharmacists participating in this study highlight their concern regarding the volume at which the robot speaks, advocating for it to have a certain degree of physical boundaries to ensure customer integrity. However, in delicate situations, as the purchasing of emergency contraception may be, it is found that boundaries should not be too obvious in nature. For example, if the robot gives counselling on only this one medicine, it being located in a separate room can make it too obvious to other customers what is being bought and thereby jeopardise customer integrity. Environmental barriers such as room dividers are frequently used in community pharmacies to increase the amount of privacy for customers.^55^ Similar customer thoughts concerning relevant barriers when receiving medication counselling by the robot to be taken into account in further development of the device are identified in the previously mentioned evaluation of potential customers experiences concerning the HRI in question.^40^

A major advantage of the social robot in medication counselling stated by the participants in this study is its benefit with respect to medication safety in situations where the customer for whatever reason feels uncomfortable about receiving counselling from a human pharmacist. Bespoke situations are such where the customer is acquainted with the pharmacists e.g., in small communities, or for some reason fears being judged. Pharmacists speculate that such issues may even lead to customers not buying the medicine. These findings are also in accordance with the study on potential customer experiences concerning the HRI,^40^ showing that the robot can offer customers a chance to maintain their self-esteem, thereby diminishing shame.

### 3.4 Interaction and robot characteristics

In scrutinising pharmacists' thoughts on the HRI and how this might affect medication safety, the appearance of the robot came up. All the participants lacked previous experience of interacting with a social robot of this kind, and reflections concerning its appearance varied widely between it making them too nervous to concentrate on the information given to the human-like appearance adding to a feeling of trustworthiness. This split in pharmacists' reactions to the robot's appearance is in line with respect to previous literature, as social robot developers have strived to create human-like qualities while simultaneously being cautious not to make them mimic human appearance too closely, with the aim of avoiding a potential unsettling feeling associated with robots that look almost human.^23^^,^^24^ At the same time, in HRI, humans and robots strive to interact on a peer-to-peer or companionable basis.^10^ In ensuring medication safety, it is therefore relevant to find the optimal balance for the social robot's appearance in a pharmacy setting.

With regard to the importance of the pharmacist being a good listener and an empathic responder in in pharmacy practice,^55^ the insights given by pharmacists regarding the high speed of the robot's speech, together with its inability to ask relevant questions and allow the customer to feel understood are highly relevant to improving and ensuring medication safety. Developing the interaction to ensure customer empowerment by securing a good enough feedback loop from the robot to the customer, for example, is of significant relevance as this has been shown to have a positive impact on customers' medication management.^56^ A major advantage expressed by the participants is the robot's ability to speak over 40 different languages since it thereby entails a desirable tool for pharmacists with respect to ensuring medication safety when working within multilingual environments, as many environments are nowadays.

### 3.5 Pharmacists' professional role and social robots

In the light of there being a shortage of personnel within community pharmacies in Finland,^5^, ^6^, ^7^ pharmacists recognise the advantages of the social robot being utilised for medication counselling. Nevertheless, they are equally concerned that they might be replaced by robots in the future, as also seen in the discussion above concerning the pharmacy context. For example, Shuaib et al. put forward the possibility of technological singularity being a hypothetical scenario in the future, as the capabilities of AI-enabled systems have increased in recent years.^57^ They state that AI in health care still needs to substantially develop artificial empathy and that there is a need to weigh up the socio-legal and ethical impacts of these systems. All the same, they conclude that the replacement of humans by AI in health care will never be absolute. According to the pharmacists in this study, an additional means to develop the social robot and make it more reliable and human-like would be to connect it to different databases giving the robot access to customer prescription history, for example. With respect to job satisfaction or dissatisfaction among pharmacists,^5^, ^6^, ^7^, ^8^, ^9^, ^10^, ^11^, ^12^ a social robot of this kind could potentially offer a remedy by freeing up pharmacists' time to concentrate on more difficult counselling tasks and professional development, and likewise diminish their workload and psychosocial burden.

Customer perceptions of pharmacists and pharmacies regarding qualities such as trustworthiness, accessibility and atmosphere may, if they are off track, cause customer-related personal barriers.^55^ Indeed, in the previously mentioned study by Gregory et al.,^46^ it is shown that trust-enhancing factors in community pharmacies include accessibility, affability, acknowledgement, respect, and interpersonal chemistry. As customer-related barriers are sometimes at play, the participants identify the social robot as a potential complement in ensuring that customers who are reluctant to receive medication counselling from a human pharmacist may accept information given by a neutral agent such as a robot. In comparison with the conferred trust as described above,^46^ earned trust builds upon interpersonal experiences over time between customer and pharmacist and is equally relevant in the further development and personalisation of the robot application.

### 3.6 Human skills and nonverbal communication

The robot's lack of body language is identified by the pharmacists as a shortcoming when compared to human beings. Also, they express that a human pharmacist can tell if a customer is lying by interpreting subtle nonverbal signs that the customer is providing; signs that they believe a robot will never be able to identify – such as facial expressions. So, both the lack of the robot's own body language and its reading of the counterpart's body language are seen as problematic. All instances of nonverbal communication cues (both conscious and unconscious) are at play and have been reported to constitute a large and essential part of our communication.^55^ Therefore, considering all the relevant elements of nonverbal communication, such as kinesics including facial expressions, proxemics and paralinguistics, are of essence in the development process.

According to the pharmacists, the social robot is not able to replace a human pharmacist, but it can offer a potential complement with respect to certain customer groups and in the light of personnel shortages. However, they emphasise that a human pharmacist must be available in every instance to ensure medication safety in case of technical or communicational issues concerning the robot. They also see the robot as a means to improve the service level in community pharmacies since it offers customers a choice in service provision, nevertheless pointing out that the choice of consulting the robot instead of a human pharmacist should always be the customers to make. Similar perspectives were found in the evaluation of potential customers' experiences of using the robot for medication counselling purposes,^40^ showing that customers value their freedom of choice in these situations depending on the nature of the situation. Sometimes they find the human pharmacist preferable, while in other cases the robot would be the one, they choose to turn to.

### 3.7 Future developments

The current study shows that, according to pharmacists, improving medication safety by providing robot medication counselling in community pharmacies comprises several different factors and practical recommendations to be considered in future developments. These include the importance of maintaining customers' trust in the pharmacy context by providing high-quality robot counselling of optimal quantity, to establish appropriate physical boundaries for the robot, to take into account customers' varying degrees of digital competency, to design a robot with an optimal appearance and feedback loop, and to consider potential effects on the professional role of pharmacists and human skills that the robot cannot currently offer. While the use of social robots in medication counselling shows the potential to offer numerous benefits, the study findings also show concerns regarding how robots can affect customers' vulnerability. To achieve an implementable robot application for medication counselling purposes, it is crucial that all aspects, both practical and sociopsychological in nature, are considered in advance.

### 3.8 Strengths and limitations

This study provides novel insights into pharmacists' views concerning the utilisation of a social robot for medication counselling purposes in community pharmacies and how this may help or harm medication safety. It illuminates the way in which medication safety can be improved by using a social robot for this purpose, adding to an important gap in the literature. From a sociotechnical point of view and as performed in this study, co-creation and development together with professionals and potential end-users is seen as beneficial for the successful implementation of technological solutions in different kinds of organisations,^58^ including pharmacies. In addition to the co-creation with pharmacy professionals and potential end-users, a broad research perspective is achieved due to the research team members' expertise within the various fields of health sciences and welfare technology, technological development, pharmaceuticals, and pharmacy practice.

Nevertheless, co-creation with pharmacy professionals may also present an obstacle with respect to study generalisability, due to the fictitious pharmacy setting used and the participants' different levels of technology experience. In addition, cross-cultural generalisability may be an issue due to the study been conducted in a single country. Other potential limitations of the study include the inductive approach used in the data analysis as this offers a risk of yielding merely general summaries and surface descriptions on the topic being investigated.^59^ However, in terms of the novelty of the research of utilising social robots for medication counselling purposes in community pharmacies, an inductive approach offers a good insight into the matter at this point as it gives depth to its understanding and guides future research endeavours in the field.

## 4 Conclusions

In summary, these findings indicate that a social robot can offer a potential complement to a human pharmacist with respect to certain customer groups and in the light of personnel shortages, but it is not able to fully replace a human pharmacist. In the future, social robots may offer an addition to the service level concerning trust, equality, freedom of choice and multilingualism in the customer service situation at community pharmacies and thereby improve medication safety.

## Funding

This work was supported by the strategic research profiling area Solutions for Health at Åbo Akademi University [10.13039/501100002341Academy of Finland, project# 336355], 10.13039/501100007247Svenska kulturfonden and Högskolestiftelsen i Österbotten.

## CRediT authorship contribution statement

**Sara Rosenberg:** Writing – review & editing, Writing – original draft, Visualization, Validation, Methodology, Investigation, Funding acquisition, Formal analysis, Data curation, Conceptualization. **Malin Andtfolk:** Writing – review & editing, Validation, Project administration, Methodology, Investigation, Funding acquisition, Formal analysis, Data curation. **Susanne Hägglund:** Writing – review & editing, Methodology, Investigation, Funding acquisition. **Mattias Wingren:** Writing – review & editing, Methodology, Investigation, Funding acquisition. **Linda Nyholm:** Writing – review & editing, Validation, Project administration, Methodology, Investigation, Funding acquisition, Formal analysis, Data curation.

## Declaration of competing interest

The authors declare the following financial interests/personal relationships which may be considered as potential competing interests.

The current research was supported by the strategic research profiling area Solutions for Health at Åbo Akademi University [10.13039/501100002341Academy of Finland, project# 336355], 10.13039/501100007247Svenska kulturfonden and Högskolestiftelsen i Österbotten.

## Appendix A Supplementary data

Interview guide. Image 1
