# Supplementary material for: Social robots counselling in community pharmacies – Helping or harming? A qualitative study of pharmacists' views
Source: Explor Res Clin Soc Pharm. 2024 Feb 24;13:100425. doi: 10.1016/j.rcsop.2024.100425 (PMC10937306; doi:10.1016/j.rcsop.2024.100425)
Supplement: Supplementary file 1 — Interview guide. [file mmc1.docx]

**Appendix A. Interview guide**

**Background questions:**

1. Age
2. Mother tongue
3. Work experience
4. Technology experience at the workplace/leisure time

**Questions regarding the interaction:**

1. Do you have previous experience with robots? In what way and in what kind of situation?
2. Do you have previous experience with similar robots that you just encountered? In what way and in what kind of situation?
3. How did you experience the interaction with the robot? Can you describe what made you experience the interaction in that way?
4. Are there advantages to using such a robot in pharmacies? What are these advantages?
5. Are there disadvantages to using such a robot in pharmacies? What are these disadvantages?

**Questions related to emergency contraception:**

1. How do you feel about the use of such robots when serving customers who buy emergency contraception? Please tell me why you think so.

2. What are your experiences of the information regarding the emergency contraception that the robot gave during the meeting? Please develop your answers.

1. If you imagine a real pharmacy meeting where this robot would be the one giving the customer counselling on the day-after pill, how would you like the meeting to look like?
2. Are you missing something in the interaction regarding the day-after pills? Feel free to discuss human/robot differences in such interactions.

**Questions regarding safety:**

1. How confidence-inspiring did you find the robot? Can you describe why? What is the reason for you experiencing this?
2. Do you think that patient safety can be guaranteed when such robots are used? How? If the answer is no, can you describe why patient safety cannot be guaranteed? Which factors threaten patient safety?

**Questions regarding future prospects:**

1. Do you see a need for such robots in pharmacies? Please elaborate on the answer.
2. What tasks do you consider suitable for such a robot in a pharmacy?
3. Which tasks do you consider less suitable for such a robot in a pharmacy?

4. Do you feel that such robots can change your profession in any way? How? Please elaborate on the answer.

5. What type of customers do you think would like to be served by such a robot in a pharmacy? Please elaborate on the answer.

1. Do you think there are customer groups for whom service by such a robot is less suitable? Please elaborate on the answer.
2. Do you feel that dignity can be preserved in the meeting between a customer and such a robot? How?
3. What would you change in the robot to make the meeting an (even) better experience?
